# Ability of *Lactobacillus brevis* 47f to Alleviate the Toxic Effects of Imidacloprid Low Concentration on the Histological Parameters and Cytokine Profile of Zebrafish (*Danio rerio*)

**DOI:** 10.3390/ijms241512290

**Published:** 2023-07-31

**Authors:** Nikita Kochetkov, Svetlana Smorodinskaya, Aleksey Vatlin, Dmitry Nikiforov-Nikishin, Alexei Nikiforov-Nikishin, Valery Danilenko, Klimuk Anastasia, Diana Reznikova, Yelena Grishina, Sergei Antipov, Maria Marsova

**Affiliations:** 1 Laboratory of Bacterial Genetics, Vavilov Institute of General Genetics, Russian Academy of Sciences, 119333 Moscow, Russia; kler.smo@gmail.com (S.S.); vatlin_alexey123@mail.ru (A.V.); niknikdl@rambler.ru (D.N.-N.); valerid@vigg.ru (V.D.); klimukanastasia27@gmail.com (K.A.); reznikova.da@phystech.edu (D.R.); grishina.e@phystech.edu (Y.G.); 2 Faculty of Biotechnology and Fisheries, Moscow State University of Technologies and Management (FCU), 73, Zemlyanoy Val Str., 109004 Moscow, Russia; 9150699@mail.ru; 3 Phystech School of Biological and Medical Physics, Moscow Institute of Physics and Technology, Institutsky Lane 9, 141700 Dolgoprudny, Russia; 4 Department of Biophysics and Biotechnology, Voronezh State University, University Square, 1, 394063 Voronezh, Russia; ss.antipov@gmail.com

**Keywords:** *Danio rerio*, detoxification, probiotic, neonicotinoids, *Lactobacillus brevis*, lactic acid bacteria, histology, histomorphometry

## Abstract

In the present article, the possible mitigation of the toxic effect of imidacloprid low-concentration chronic exposure on *Danio rerio* by the probiotic strain *Lactobacillus brevis* 47f (1 × 10^8^ CFU/g) was examined. It was found that even sublethal concentration (2500 µg/L) could lead to the death of some fish during the 60-day chronic experiment. However, the use of *Lactobacillus brevis* 47f partially reduced the toxic effects, resulting in an increased survival rate and a significant reduction of morphohistological lesions in the intestines and kidneys of *Danio rerio*. The kidneys were found to be the most susceptible organ to toxic exposure, showing significant disturbances. Calculation of the histopathological index, measurement of morphometric parameters, and analysis of principal components revealed the most significant parameters affected by the combined action of imidacloprid and *Lactobacillus brevis* 47f. This effect of imidacloprid and the probiotic strain had a multidirectional influence on various pro/anti-inflammatory cytokines (IL-1β, TNF-α, IL-6, IL-8). Therefore, the results suggest the possibility of further studying the probiotic strain *Lactobacillus brevis* 47f as a strain that reduces the toxic effects of xenobiotics. Additionally, the study established the possibility of using imidacloprid as a model toxicant to assess the detoxification ability of probiotics on the kidney and gastrointestinal tract of fish.

## 1. Introduction

Probiotic supplements are actively used in agriculture, including aquaculture, to modulate immunity [1], enhance disease resistance [2,3], and improve the feed conversion ratio by influencing nutrient metabolism and energy homeostasis [4,5]. Probiotics are microorganisms that have a positive effect on the overall health of the host [6]. Bacteria from the genera *Bacillus*, *Lactobacillus*, and *Bifidobacterium* are the most commonly used in industrial aquaculture [6,7,8,9,10]. Some of these microorganisms are capable of becoming established in the intestinal microbiota of aquatic organisms, and their metabolites and cellular components contribute to the stabilization of the commensal microbial community, influencing host physiological processes [11,12].

The application of probiotics to reduce or alleviate the acute and chronic toxic effects of various pollutants has garnered significant research interest [13,14]. Several studies have demonstrated that probiotic bacteria can bind and/or metabolize different chemical pollutants, particularly heavy metals, organophosphate pesticides, and mycotoxins [15,16,17,18]. Furthermore, probiotics have been shown to be a safe alternative to antibiotics and other chemotherapeutants [19].

In this regard, the search and characterization of new strains of probiotic microorganisms for use as functional food and/or feed additives is currently being actively pursued [20]. The laboratory fish *Danio rerio* can serve as a convenient alternative for preliminary investigation of the beneficial properties of probiotics. Zebrafish are actively used as a model organism to study various human diseases [21], ecotoxicological effects [22], pharmacological studies [23], and the composition and changes in the gut microbiome [24]. Studies performed on *Danio rerio* provide a cost-effective assessment of the combined effects of a toxicant and a probiotic on various biomarkers, such as the histological structure of organs and tissues, hematological parameters, and the expression of immune response and detoxification markers. The data obtained from such studies, including probiotic concentrations, mechanisms of action, and possible limitations in their use, can be partially extrapolated to other animal species and humans [25].

It is advisable to conduct prolonged studies during the study of xenobiotics detoxification since pollutants are most often found in extremely low concentrations in the environment, and their continuous exposure leads to chronic intoxication, which differs from acute toxicity not only in terms of duration but also in the intensity of the toxicant’s effects [26]. Additionally, probiotic bacteria may not always establish themselves as part of the commensal microbiome, and a long-term experiment will provide an opportunity to more comprehensively evaluate the interactions between xenobiotics, probiotics, and the host [5].

The effects of sublethal concentrations of toxicants should be determined not only by assessing mortality, growth/mass, and reproductive parameters but also by utilizing more sensitive indicators [27]. Among these indicators, hematological, biochemical [16,28], ethological [29], molecular biological techniques [30], and histological examination are the most commonly employed. Histological studies of aquatic organisms are widely utilized in toxicological research to assess the impact of toxicants on fish health [31,32,33]. They are also employed in aquaculture studies investigating the effects of new feed recipes and feed additives on the gastrointestinal tract [34,35,36]. The key advantages of studying histological structures are as follows: (i) high sensitivity to different types of treatment/exposure, (ii) ability to identify the most affected organs (target organs), (iii) determination of lesion dynamics, enabling the establishment of concentration/time-effect relationships, and (iv) description of histological lesions, which can be used as qualitative or semi-quantitative indices in the subsequent analysis [37,38]. Components of the fish gastrointestinal tract (GIT) are primarily affected by the use of various functional supplements in feed, as the GIT represents a key pathway for substance intake, digestion, and nutrient absorption [39]. Upon entering the organism, xenobiotics can disrupt the barrier function of the intestinal mucosa, interfere with biochemical and immune processes, impact nutrition, and alter the composition of the microbiota [40]. Therefore, it is important to focus on the organs involved in resorption (intestine), biotransformation (liver), and excretion (kidneys/gills) of the toxicant during the study of the detoxification properties of probiotics.

Imidacloprid (IMI) is a member of the neonicotinoids (NEOs) family of neuroactive insecticides modeled after nicotine [41]. Neonicotinoids, including mixtures with other active ingredients, are widely used in agriculture, medical, sanitary, and household disinsection to control pests and synanthropic insects. As cholinergic toxicants and agonists of postsynaptic nicotinic acetylcholine receptors, NEOs are much more toxic to insects [42]. IMI has been found to have immunotoxic [43], neurotoxic [44], mutagenic, and teratogenic effects on mammals [45]. The entry of IMI into aquatic ecosystems in agricultural regions through runoff can have toxic effects on hydrobionts [46,47]. Moreover, NEOs, such as IMI, as systemic insecticides, can be absorbed into plant tissues, released into the air with pollen, and diffused into the soil, water, and agricultural products. NEOs and their metabolites have been detected in human urine, saliva, serum, and breast milk [48,49]. It has been shown that low doses/concentrations of imidacloprid, especially with chronic exposure (100–2000 µg/L), can lead to metabolic disturbances [50], impaired gut barrier function [51], and liver and kidney dysfunction [52]. Therefore, the use of IMI as a model toxicant is relevant in the study of the detoxification effect of probiotics.

The bacterial strain *Lactobacillus brevis* 47f was selected from the Lactobacillus collection for its pronounced antioxidant [53] and adaptogenic properties [54]. It has demonstrated high activity in reducing oxidative stress and maintaining the normal histological structure of the mouse gut [55]. While most probiotics used in aquaculture are obtained from terrestrial animals or other sources [56], some authors suggest that autochthonous (host-associated) probiotics are more suitable for incorporation into fish feeds as they are better adapted to the host immune system and possess the necessary combination of digestive enzymes and bioactive compounds [20,57,58]. Nevertheless, many lactic acid bacteria (LAB) of allochthonous (external) origin can also be potentially applied in fish due to their high adhesive capacity and resistance to acidity and bile acid salts [59]. Based on previous studies and data on the application of LAB, we hypothesize that the strain *Lactobacillus brevis* 47f will retain its activity at lower temperatures and the aerobic gut conditions, typical for poikilothermic animals, such as fish.

Thus, the aim of this study is to determine the effects of *Lactobacillus brevis* 47f on the histological parameters of the intestine, liver, and kidneys of *Danio rerio* under exposure to low doses of imidacloprid in a chronic experiment. In addition, the effects of the strain on the expression of intestine genes responsible for the immune response were evaluated.

## 2. Results

### 2.1. Survival Rate and Weight Gain

**Figure 1:**
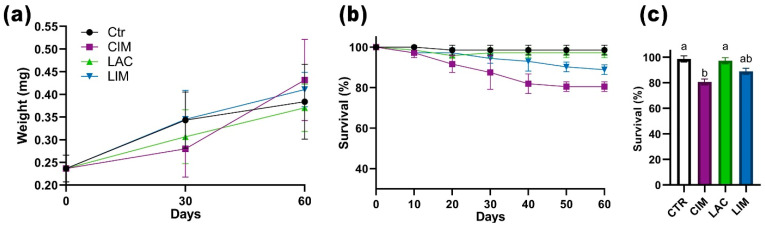
Survival dynamics (**b**), 60-day survival (**c**), and weight (**a**) of *Danio rerio* when exposed to imidacloprid solution. Value (*p* < 0.05) from the Kruskal–Wallis test. The letters above each bar (a, b) show statistical significance between the different experimental groups.

Exposure of *Danio rerio* to imidacloprid (IMI) solutions resulted in a significant decrease in the survival rate in the positive control group (CIM; *p* < 0.05), which was 80.5% on day 60 of the experiment (Figure 1c). In the group treated with probiotic feed and exposed to imidacloprid (LIM), the survival rate was 88.8% (Figure 1b). No significant differences were found between the other experimental groups.

### 2.2. Intestinal Histology

**Figure 2:**
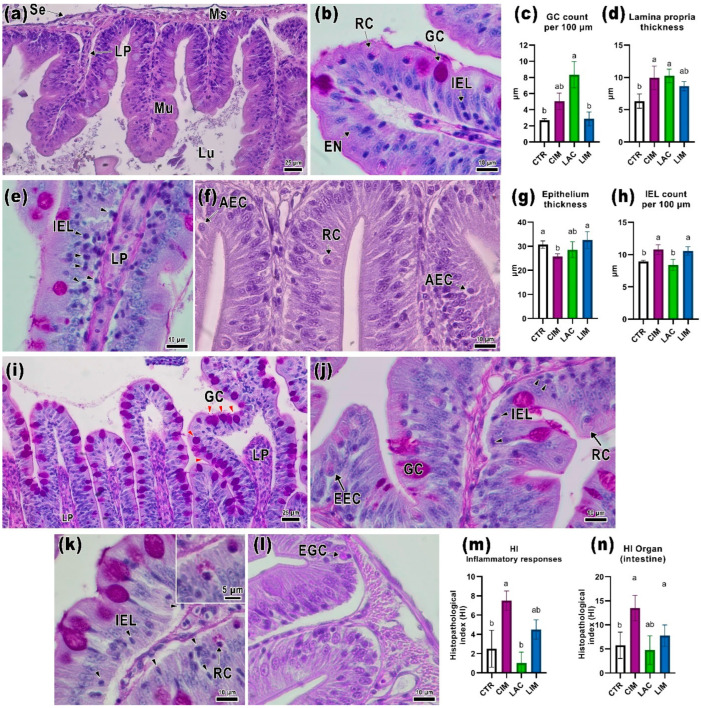
Transversal histological sections of the intestine of *Danio rerio* in control and experimental groups and morphometric parameters (**c**,**d**,**g**,**h**), histopathological index (**m**,**n**). CTR (**a**,**b**): normal histology of the intestines of control fish, including three layers: lumen (Lu) serous membrane (Se), muscular layer (Ms), and mucous layer (Mu) arranged on the lamina propria (LP). The mucosa consists of epithelial cells with oval nuclei (EN), goblet cells (GC), PAS-positive Rodlet cells (RC), and intraepithelial leukocytes (IEL). CIM (**e**,**f**): intraepithelial leukocyte accumulation (black arrowheads) and lamina propria enlargement can be seen in the intestine of the negative control. Apoptotic epithelial cells (AEC) are observed in some areas of the mucosa. LAC (**i**,**j**): an increase in the number of goblet cells (red arrow) and enlargement of the lamina propria are observed in the intestine when the probiotic is used. Enteroendocrine cells (EEC) and Rodlet cells are also seen in the mucosa. LIM (**k**,**l**): the combined effect of the probiotic and the toxicant is expressed by an increase in the number of intraepithelial leukocytes and the presence of eosinophilic granulocytes (EGC). H&E (**a**,**f**,**l**) and PAS (**b**,**e**,**i**,**j**,**k**) staining. Scale bar: 25 μm (**b**,**e**,**f**,**j**–**l**) and 10 μm (**a**,**i**). Significance (*p* < 0.05) from the Kruskal–Wallis test. The letters above each bar (a, b) indicate statistical significance between the different experimental groups.

Figure 2a,b shows transverse sections of the intestine of *Danio rerio* in the control group, where several layers can be distinguished: the serous membrane, muscle layer, and mucous layer. The villi, formed by extensions of the mucosa, are covered with columnar adsorbing epithelium, goblet cells, and other enteroendocrine cells (e.g., Rodlet cells). In the center of the villi, there is the lamina propria, which consists of loose connective tissue and a vascular network. In the basal part of the mucosal epithelium, it is also possible to see groups of intraepithelial leukocytes with pronounced basophilic staining. In the control group, their number was 8.9 ± 0.16 cells per 100 µm of mucosa (Table S2).

In the CIM group, there were clusters of intraepithelial leukocytes throughout the mucosa (Figure 2e). Their number was significantly higher than in the CTR and LAC groups (*p* < 0.05; Figure 2h). There was also a significant increase in the thickness of the lamina propria in this group compared to the control group (*p* < 0.05; Figure 2d). Cells with signs of apoptosis (McKnight cells) were found in some areas of the epithelium (Figure 2f). Additionally, the presence of a large number of Rodlet cells in the mucosa should be noted. Rodlet cells were oval cells with a formalized nucleus, located near the apical part of epithelial cells, containing eosinophilic rods or granules inside. The height of the adsorbing epithelium in the CIM group was significantly lower than in the control and LIM groups (Figure 2g).

The intestines of the group of fish fed the probiotic *Lactobacillus brevis* 47f (LAC) showed a number of morphological deviations from the control. The number of goblet cells per 100 µm of epithelium was significantly higher than in the CTR and LIM groups (*p* < 0.05; Figure 2c,i) and reached 8.3 ± 1.4. The thickness of the lamina propria was significantly higher than in the control group (*p* < 0.05; Figure 2d), and there were no significant differences in the number of intraepithelial leukocytes. The mucosa of the villi in this group also showed a significant number of Rodlet cells and enteroendocrine cells, most often located near the clusters of intraepithelial leukocytes (Figure 2j).

In the mucosa of the LIM group, the number of intraepithelial leukocytes was significantly higher than in the CTR and LAC groups (*p* < 0.05; Figure 2h). No differences in other morphometric parameters were detected. Accumulations of intraepithelial leukocytes and an increase in the number of goblet cells were found in certain sections of intestinal slides (Figure 2k). A few eosinophilic granulocytes were also found at the base of the villi (Figure 2l).

In the CIM group, the development of an inflammatory response in the intestine (HI_irI_) was detected, namely: enlargement of the lamina propria, an increased number of intraepithelial leukocytes, and the presence of eosinophilic granulocytes. This response pattern significantly differed in the CIM group from the control and probiotic group (*p* < 0.05; Figure 2m; Table S1). According to the data obtained, the value of the total intestinal histological index (HI_ioI_) was significantly higher in the CIM group (*p* < 0.05; Figure 2n).

### 2.3. Liver Histology

**Figure 3:**
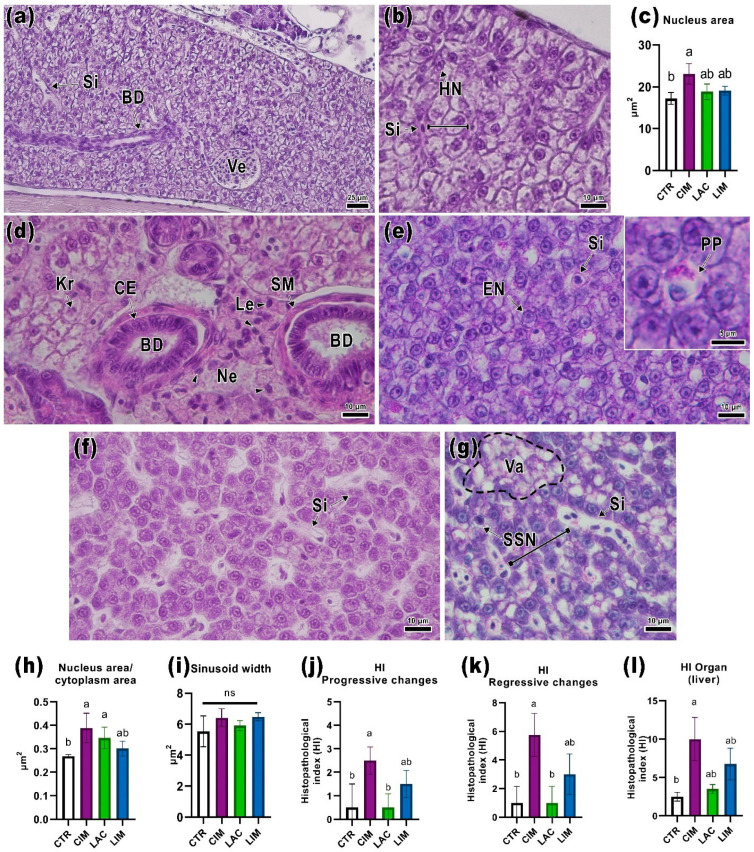
Histological sections of the liver of *Danio rerio* in control and experimental groups and indices of morphometry (**c**,**h**,**i**), histopathological index (**i**,**j**,**l**). CTR (**a**,**b**): normal liver structure of control *Danio rerio*. Sinusoids (Si) dividing hepatocytes (HN) into anastomotic lobules (black line), as well as large capillaries (Ve) and bile ducts (BD). CIM (**d**,**e**): exposure to IMI resulted in foci of necrosis (Ne) and cells with cariorexis (Kr) associated with leukocyte infiltration (Le, black arrowheads). PAS-positive granules in the cytoplasm of hepatocytes (PP). Hepatocytes with enlarged nuclei (EN) were also observed in the liver. The normal organization of the bile ducts, including a smooth muscle sheath (SM) and cubital epithelium (CE), was noted. LAC (**f**): a section of the liver with normal hepatocyte organization and slightly dilated sinusoids. LIM (**g**): combined treatment with the probiotic and IMI resulted in increased vacuolization (Va, dotted line) and the number of single-cell necrosis (SCN) and slightly dilated sinusoidal capillaries. H&E staining (**a**,**b**,**d**,**f**) and PAS (**e**,**g**). Scale bar: 25 μm (**a**) and 10 μm (**b**,**d**–**g**). Significance (*p* < 0.05) from the Kruskal–Wallis test. The letters above each bar (a, b) indicate statistical significance between the different experimental groups. ns: *p* > 0.05.

The livers of control animals (CTR) included mono- and binuclear hepatocytes divided into anastomotic lobules (cords) by sinusoidal capillaries (Figure 3b). Hepatocytes had basophilic staining and signs of vacuolization, probably due to the presence of glycogen/fat inclusions. Large vessels and bile ducts were also found in the organ parenchyma (Figure 3a). No significant pathological abnormalities were found in the CTR group.

In the liver of the CIM group, foci of necrosis located near the bile ducts were detected (Figure 3d). Cells with cariorexis were found in these areas as well as leukocyte infiltration. Bile duct morphology was normal. Measurement of histomorphological parameters showed that the nucleus area and nucleus/cytoplasm area ratio significantly differed from the control group (*p* < 0.05; Figure 3c,h). PAS-positive granules were observed in the cytoplasm of some hepatocytes (Figure 3e).

The use of a probiotic supplement (LAC) had no significant effect on the structure of the liver. There was a slight increase in the width of the sinusoids in some parts of the organ (Figure 3f,i).

Similar changes were also noted in the LIM group. In the liver of this group, an increase in vacuolization located in foci was found (Figure 3g). Separately, the presence of cells with single-cell necrosis detected in all individuals of this group should be noted. Irregular histoarchitectonics of the organ in the LIM group, expressed in changes in the distance between the anastomotic lobules, is probably related to the above-described hepatocellular changes.

Calculating the histopathological index of the liver, regressive disorders (HI_rcL_) were the response pattern that had the greatest impact on the function of the organ. In the CIM group, the value of this index was significantly higher than in the control group (*p* < 0.05; Figure 3k) due to the presence of necrosis foci near the bile ducts. The enlarged hepatocyte nuclei in this group resulted in a non-significant increase in the pattern of progressive disorders (HI_ioL_, Figure 3i). The histopathological index of the organ (Figure 3j) in the positive control (CIM) was significantly higher than in the CTR and LAC groups (*p* < 0.05).

### 2.4. Kidney Histology

**Figure 4:**
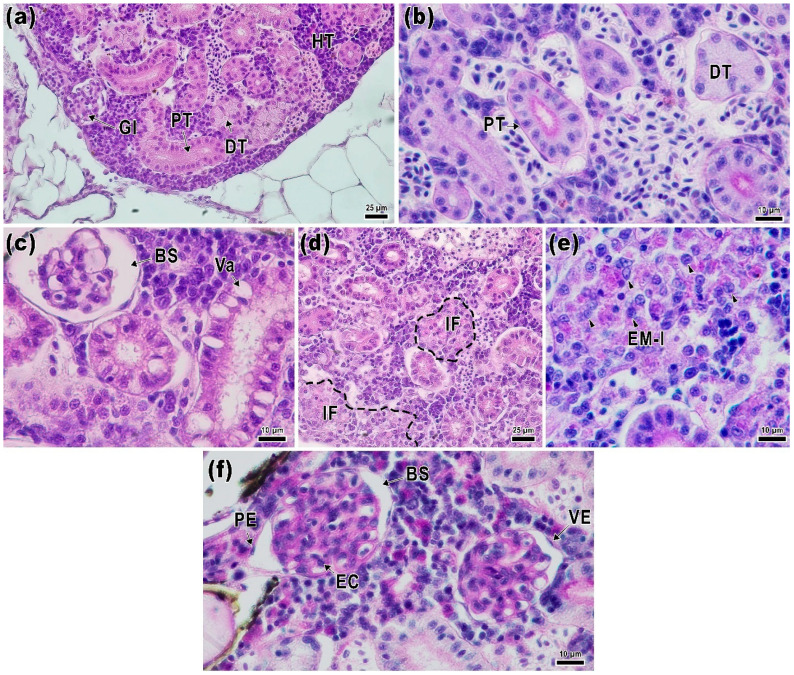
Histological sections of the kidney of *Danio rerio* control and experimental groups. CTR (**a**,**b**): normal kidney morphology in the control animals. The kidney includes a glomerulus (Gl) located within the renal corpuscle, as well as distal (DT) and proximal (PT) tubules surrounded by hematopoietic tissue (HT). The distal tubules exhibit a pronounced basophilic cytoplasmic straining and a brush border. Proximal tubules have chromophilic cytoplasm, and their nuclei are shifted towards the basal part. CIM (**c**,**d**,**e**): vacuoles (Va) appear in the tubule epithelium under the impact of IMI, and an increase in Bowman’s space (BS) is observed. Focal foci of inflammation (IF, dotted line) contain nuclear and cytoplasmic debris, mononuclear infiltrate, and macrophages, which are probably epithelioid macrophages (EM-l, black arrowheads) with large nuclei. LAC (**f**): normal renal structure in the LAC group, where renal corpuscles with endothelial cells (EC), as well as visceral (VE) and parietal epithelium (PE) can be distinguished. H&E (**a**,**c**,**d**) and PAS (**b**,**e**,**f**) staining. Scale bar: 25 μm (**a**,**d**) and 10 μm (**b**,**c**,**e**,**f**).

The kidney of the control fish exhibited the typical morphology for this species [60]. Hematopoietic tissue is situated within the stroma of the organ, encompassing a variety of precursors for blood-forming elements, along with renal corpuscles and tubules. The renal corpuscle is enclosed by the parietal epithelium and separated from the glomerulus by Bowman’s space. The glomerulus, separated from the space by the visceral layer, comprises endothelial and mesangial cells (Figure 4a,b).

The epithelium of the proximal tubule exhibited eosinophilic cytoplasm staining and a pronounced PAS-positive brush border (Figure 4b). The distal part, located furthest from the glomerulus, consisted of cube-shaped cells with weakly stained cytoplasm, and oval nuclei displaced towards the basal membrane, and did not exhibit a distinct brush border as observed with PAS staining. Comparatively, the basal membrane of the collecting ducts showed more pronounced staining than the distal and proximal tubules (Figure 4b).

**Figure 5:**
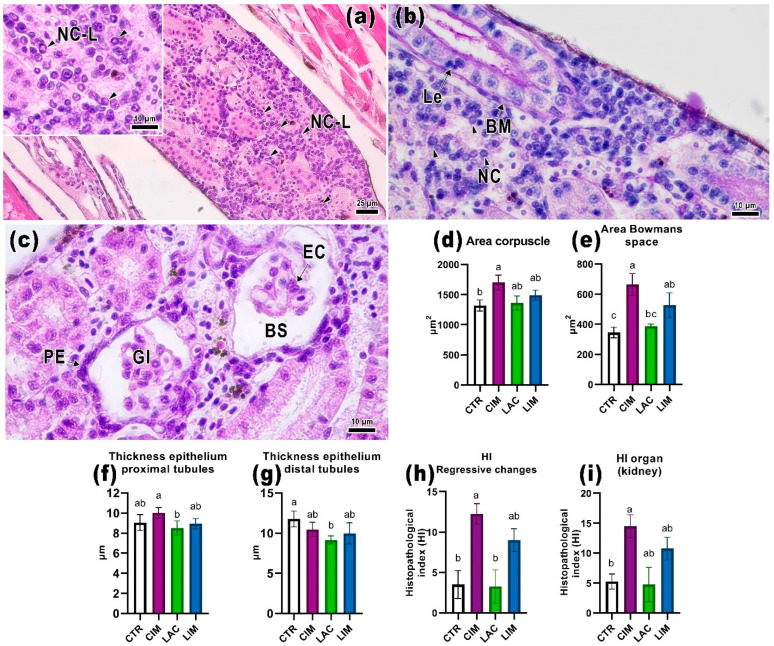
Histological sections of *Danio rerio* kidneys from the LIM group, morphometric parameters (**d**–**g**) and histopathological indices (**h**,**i**). LIM (**a**,**b**): Presence of necrotic-like cells (NC-L, black arrowheads) in the hematopoietic tissue, displaying apoptotic nuclei with amphophilic staining. Thickening of the basal membrane (BM) and presence of leukocytes (Le) in the renal tubule epithelium can also be observed. (**c**) Degeneration of renal tubules with subtle pleomorphism of endothelial cells (EC) and enlargement of Bowman’s space (BS). Thickening of certain areas of the parietal epithelium (PE). H&E staining (**a**,**c**) and PAS staining (**b**). Scale bar: 25 μm (**a**) and 10 μm (**b**,**c**). Significance (*p* < 0.05) was determined using the Kruskal–Wallis test. The letters above each bar (a, b) indicate the statistical significance between the different experimental groups.

In the CIM group, there was a disruption of the normal renal structure characterized by vacuolization of the cuboidal epithelium of the proximal tubules and an increase in Bowman’s space (Figure 4c). There was a significant enlargement of Bowman’s space (*p* < 0.05) and, consequently, an increase in the renal corpuscle area (*p* < 0.05; Figure 4d,e). The proximal tubule epithelium also exhibited a significantly greater thickness compared to the LAC group (Figure 4f). Additionally, focal inflammatory foci were observed (Figure 4d). Apart from mononuclear leukocytes, these foci contained epithelioid macrophage-like cells with large, formalized nuclei exhibiting basophilic staining. PAS-positive staining revealed cell debris within the inflammatory areas (Figure 4e). In the LAC group, the distal tubule thickness was significantly lower than in the control group (Figure 5g).

In the LIM group, several pathological abnormalities were observed in the kidney structure. All examined samples exhibited signs of necrosis in the areas containing hematopoietic tissue, confined to small focal areas. Cells in these regions displayed apoptotic nuclei with amphophilic staining (Figure 5a). Lesions in the renal corpuscles were characterized by glomerular degradation (a significant increase in Bowman’s space compared to CTR, *p* < 0.05; Figure 5e), thickening of the parietal epithelium, and nuclear pleomorphism of the endothelial cells in some nephrons (Figure 5c).

The main histopathological response induced by IMI in the kidneys was regressive changes (HI_rcK_), which were significantly higher in the CIM group compared to the control group (*p* < 0.05; Figure 5h). Histological alterations in the positive control (CIM) primarily manifested as vacuolization of tubule epithelium, enlargement of Bowman’s space, and the possible presence of inflammatory foci in the hematopoietic tissue. Fish in the CIM group exhibited a higher value (*p* < 0.05; Figure 5i) for the kidney histopathological index (HIki) compared with the control group. However, no significant differences were observed in the LAC group.

### 2.5. Principal Component Analysis

**Figure 6:**
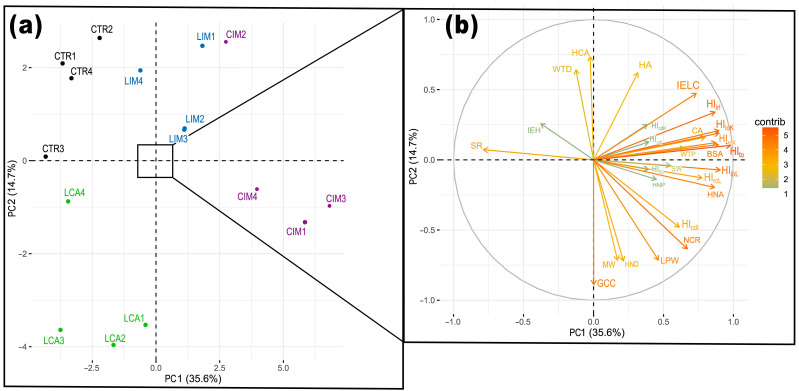
Principal component analysis of histopathological indices and morphometric parameters in intestine, liver, and kidney of *Danio rerio* (**a**). Abbreviations are presented in materials and methods. Graph (**b**) shows the indexes/parameters with the highest contribution.

PCA of the histopathological index data and morphometric parameters of the intestine, liver, and kidney confirmed the beneficial effect of *Lactobacillus brevis* 47f in mitigating the toxic effects of imidacloprid. Two principal components (PC1 and PC2) accounted for 50.6% of the total data distribution (Figure 6). Significant differences were observed between the CTR, LAC, CIM, and LIM groups. The toxic effects of imidacloprid were manifested in changes in histological indexes of intestinal inflammatory response (HI_irL_), renal regression changes (HI_rcK_), and liver regression changes (HI_rcL_), as well as morphometric indexes such as Bowman’s space area (BSA), intraepithelial leukocyte count (IELC), and hepatocyte nucleus area (HNA). Differences in the toxicity of imidacloprid with and without the use of the probiotic were observed. In the LIM group, the toxic effects of imidacloprid were evident in changes in hepatocyte area (HA), hepatocyte cytoplasmic area (HCA), and distal tubule wall thickness (WTD). *Lactobacillus brevis* 47f primarily influenced the number of goblet cells (GCC) in the intestinal tissue.

### 2.6. qRT-PCR Analyzes of Immune-Related Genes in Intestine

**Figure 7:**
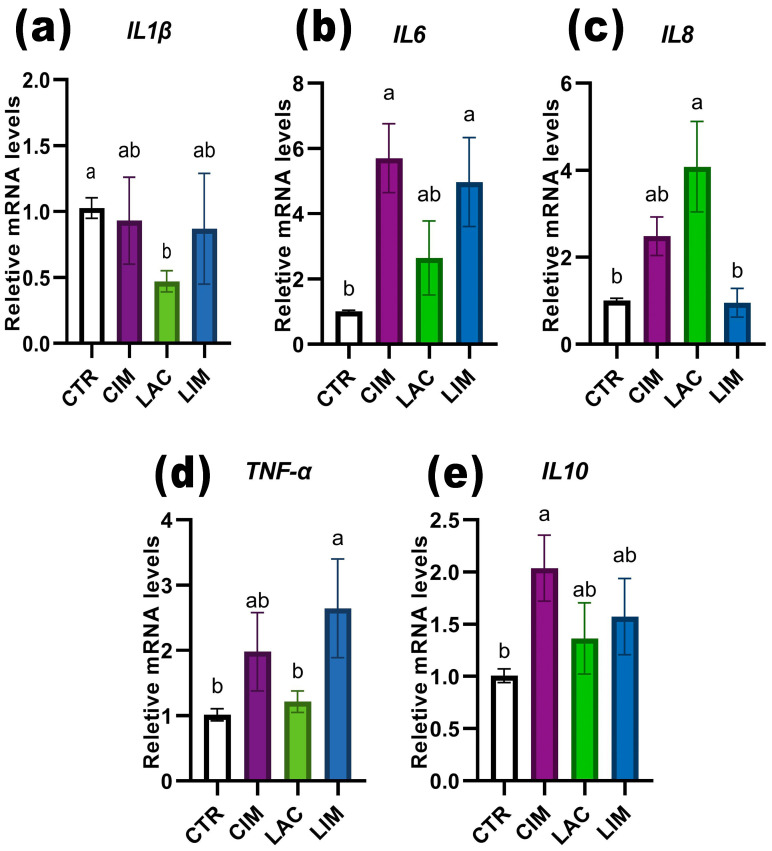
Changes in the relative expression levels of the proinflammatory genes: interleukin 1 beta, tumor necrosis factor a, interleukin 6, interleukin 8, and the anti-inflammatory gene interleukin 10 in the intestine of *Danio rerio*. Significance (*p* < 0.05) was determined using the Kruskal–Wallis test. The superscript letters (a, b) indicate the statistical significance between the different experimental groups.

Exposure of *Danio rerio* to an IMI solution led to a significant increase (*p* < 0.05) in the levels of the proinflammatory immune cytokines IL-6 and IL-10 compared to the control and LAC groups (Figure 7b,e). The LIM group also exhibited a significant upregulation of IL-6 and TNF-α levels (Figure 7b,d). However, when a probiotic strain was included in the feed, there was a significant (*p* < 0.05) decrease in the relative amount of IL-1β mRNA (Figure 7a) and an increase in IL-8 (Figure 7c). It is worth noting that the expression levels of the IL-6, IL-8, and IL-10 genes in the LIM group were slightly lower than those in the CIM group, which was exposed to IMI but did not receive the probiotic with the feed.

## 3. Discussion

### 3.1. Toxic Effects of Sublital Concentrations of Imidacloprid

The widespread use of neonicotinoids as pesticides in modern agriculture has resulted in significant contamination of water systems [41]. Imidacloprid and several other compounds have been reported to be present in low concentrations in natural water bodies and soil [50,61,62,63], ranging from 100 to 2000 µg/L. Although these concentrations are considered safe for most aquatic organisms, prolonged exposure to the chemical and its metabolites can lead to adverse effects. Luo et al. [51] found negative effects on the gut morphology and the composition of commensal microbiota of *Danio rerio* exposed to IMI at a concentration of 1000 µg/L. Similarly, exposure of *Prochilodus lineatus* to concentrations of up to 1250 µg/L IMI resulted in increased oxidative stress and oxidative DNA damage [52]. In addition, it should be noted that IMI resulted in significant disorders of renal tissue, manifested in degradation and necrosis of renal tubules, as well as gill damage in *Oreochromis mossambicus* and *Labeo rohita* [64]. Many pesticides, including IMI, have endocrine-disruptive properties, which can affect the reproductive function of fish [65], as well as disrupt the regulation of immunity [66]. The aforementioned studies, along with the results obtained in this work, confirm the systemic toxic effect of low concentrations of IMI.

In the present study, it was demonstrated that chronic exposure to IMI at a concentration of 2500 µg/L significantly increased the mortality of *Danio rerio* at 60 days without causing changes in weight values. Commercial preparations incorporating IMI are more toxic than the analytical grade compound due to the presence of other compounds that can have additive effects [60]. It is likely that prolonged exposure (60 days) to IMI resulted in cumulative toxic effects, manifested by histopathological abnormalities in renal tissue, which potentially led to fish mortality. The inclusion of the probiotic strain *Lactobacillus brevis* 47f in the feed during IMI exposure in the LIM group contributed to the emergence of adaptive reactions that reduced fish mortality, as indicated by the normalization of renal tissue.

### 3.2. Histological Parameters of the Intestine

Studies of the histological parameters in experimental *Danio rerio* individuals allowed us to identify the main lesions induced by IMI and the effects of the probiotic strain. The intestine is a complex organ involved in various functions such as digestion, nutrient absorption, electrolyte balance, immune response, and endocrine regulation of metabolism [67]. A notable abnormality specific to the IMI (CIM and LIM) groups is the increased number of intraepithelial leukocytes in the mucosa. These cells primarily consist of leukocytic lineage elements and belong to the gut-associated lymphoid tissue (GALT) [68]. Additionally, granular eosinophils, sometimes found near the muscle layer and lamina propria of the intestines of experimental fish, can also be associated with GALT. The main function of this cell group is to neutralize/eliminate stressors and promote tissue repair [69].

In the examined histological preparations of *Danio rerio*, intraepithelial leukocytes were predominantly located near the basal membrane of mucosal epithelial cells, exhibited basophilic staining, and had an oval or round shape with indistinct edges, which is consistent with previous descriptions [70]. Numerous studies on different fish species have demonstrated that changes in feed [25,71], various feed additives (e.g., probiotics [72,73,74]), and certain toxicants [30,75] can lead to an increase in leukocytic cells in the intestinal mucosa. However, there was no significant increase in intraepithelial leukocytes in the LAC group, but there was a significant increase in lamina propria. It can be concluded that IMI has an effect on intestinal immunity, while the inclusion of *Lactobacillus brevis* 47f in the diet does not have a significant effect on the immobilization of leukocytic cells.

In addition to goblet cells, the presence of other endocrine cells was observed in the intestines of *the Danio rerio*. In cases where a cell could not be clearly categorized as one of the known types (e.g., goblet cell or Rodlet cell), it was referred to as an enteroendocrine cell (EEC), which showed a slight variation in occurrence among the experimental groups. In the experimental groups, an increase in the number of Rodlet cells was found in certain areas of the mucosa. These cells are considered to be secretory cells of the holocrine type [76] and have an osmoregulatory function [77]. According to Reite [78], they may also be involved in the host defense system, along with eosinophilic granulocytes. Previous studies have shown that toxic exposure can lead to an increase in the number of Rodlet cells [79]. It can be assumed that Rodlet cells, along with goblet cells, respond to toxic compounds and the inclusion of probiotic bacteria in the feed. Future studies could focus on the presence of these cells in the intestinal mucosa and other organs, as well as changes in their numbers in response to toxicants or food/feed additives.

### 3.3. Histological Parameters of the Liver

The liver is a vital organ responsible for numerous functions, including protein, lipid, and carbohydrate metabolism, bile formation, and detoxification [80,81]. Additionally, the liver serves as a storage site for various substances, primarily glycogen and lipids. The histological structure of the organ observed in this study was consistent with previous descriptions for *Danio rerio* and other fish species [60,81,82]. The abnormalities detected in the CIM and LIM groups, such as foci of necrosis near the bile ducts, cells with cariorexis, small foci of vacuolization, and disruptions in the organ’s architecture, along with morphometric changes, are likely associated with increased detoxification in response to the presence of toxic compounds [39]. For instance, hepatocyte hypertrophy is commonly linked to toxicant exposure [83], and an increase in the nucleus/cytoplasm area ratio was observed in the CIM group. However, it is noteworthy that the group of fish receiving the probiotic with their food exhibited less severe histological changes and lower values of morphometric parameters. Since IMI can undergo biotransformation in fish, leading to the formation of hydroxyl-imidacloprid and other compounds [50], the release of these products, along with bile, could cause damage to hepatocytes near the bile ducts, as observed in the CIM group.

### 3.4. Histological Parameters of the Kidneys

The kidneys are composed of functional units called nephrons, which are responsible for metabolite excretion, osmoregulation, and maintenance of acid-base balance in the body [84]. Noteworthy histopathological abnormalities observed in the kidneys of the CIM and LIM groups include structural abnormalities in the glomeruli and necrosis of hematopoietic tissue. Since the kidneys, along with the gills, are the primary route of xenobiotic (metabolite) excretion in fish, they are highly susceptible to toxic effects [23,32]. The high values of regressive changes observed in the experimental groups with IMI supplementation suggest a degradation of renal tissue in the fish [85]. The increased thickness of the proximal nephron in the CIM group may be attributed to the presence of IMI or its metabolites in the filtrate, as the proximal nephron is responsible for the majority of reabsorption function [86]. The presence of foci of hematopoietic tissue inflammation in this group, without a clearly distinguishable peripheral membrane, along with the presence of epithelioid macrophages, may indicate granulomatous inflammation [87]. Abnormalities, such as vacuolization of the tubule epithelium, enlargement of the Bowman space, and foci of inflammation and necrosis of hematopoietic tissue, can significantly impair renal filtration function. Similar kidney structural changes were observed in *Oreochromis mossambicus* and *Labeo rohita* exposed to sublethal concentrations of IMI [87]. The histopathological changes noticed in the kidneys of the CIM group can potentially explain the mortality recorded in this experiment.

Individual hematopoietic tissue cells displaying signs of necrosis (e.g., a large amount of euchromatin, amphophilic staining, and surrounded by free space) found in the kidneys of the LIM group can occur in a healthy kidney [86]. The use of *Lactobacillus brevis* 47f slightly reduced the number of histological abnormalities, primarily observed in the renal corpuscles, and resulted in a decrease in the histopathological index. In Zang et al. [30], the *Lactobacillus plantarum* ST-III strain also demonstrated a mitigating effect on the toxic effects of triclosan on the kidneys.

### 3.5. Expression of Intestinal Pro/Anti-Inflammatory Genes

The effects of IMI and *Lactobacillus brevis* 47f on the expression levels of certain pro/anti-inflammatory immune cytokines in the intestine of *Danio rerio* were investigated. Cytokines are protein mediators produced by immune cells in the body and contribute to the growth, differentiation, and activation of defense mechanisms. IL-6 plays a crucial role in maintaining immune homeostasis, particularly in inflammation and antibody production by B-lymphocytes and cytotoxic T-lymphocytes [88]. On the other hand, IL-10 is an anti-inflammatory cytokine produced to prevent the development of an inflammatory response and activate repair processes [89]. The relative mRNA levels of IL-6, IL-10, and TNF-α were significantly altered in the groups exposed to IMI. Noteworthy is that the expression of proinflammatory cytokines can correlate with the acute stage of the inflammatory response [90], which is more characteristic of acute exposure to toxicants. In chronic low-concentration experiments, the maximum levels of their expression were related to the initial stages of toxic tissue damage and the development of the inflammatory process. Previous studies have shown contradictory effects of IMI on the expression levels of proinflammatory cytokines (IL-1β, TNF-α, IL-6, IL-8), either reducing their expression [91] or leading to upregulation [51]. This study also demonstrated that IMI has a multidirectional effect on various pro/anti-inflammatory cytokines. Thus, the expression of IL-1β and IL-8 in the IMI-exposed groups was at the same level as in the control, while IL-6 was significantly up-regulated. These results may indicate that IMI induces the development of a pro-inflammatory response in the intestine of *Danio rerio*, which is not prolonged, as IL-6 may be involved in the resolution of inflammation and initiation of the immune response [92]. This is also indicated by an increase in the relative expression of IL-10 in the CIM group.

IL-8, which belongs to the group of pro-inflammatory chemokines, can attract and activate leukocytes and serve as a mediator of inflammation [93,94]. The significant increase in its expression in the LAC group is partially supported by histological data and suggests that IL-8 regulation is improved with *Lactobacillus brevis* 47f. Previous studies have consistently shown that probiotic bacteria play a significant role in regulating the host immune response [95], and their supplementation in feed leads to increased expression of proinflammatory cytokines [12,20,30,73]. Some authors note that the reduction/inhibition of proinflammatory gene expression depends on the strain of the probiotic organism [96]. The main pathway of inhibiting proinflammatory cytokines by probiotics is presumably through the ubiquitin-proteasome protein degradation pathway (UPP) [97], and modulation via activation of the toll-like receptor 4 (TLR4) signaling pathway [98].

The decrease in IL-8 levels in the LIM group probably indicates that IMI and *Lactobacillus brevis* 47f are involved in the regulation of the expression of this chemokine, and their combined action leads to the normalization or modulation of the innate immune response of fish at the cellular and molecular levels. Some studies have shown that low concentrations of toxicants lead to an increase in the expression levels of genes associated with the immune system, while high concentrations inhibit them [99,100]. Probably, a similar antagonistic effect is also manifested in the simultaneous application of low doses of toxicants and probiotics.

### 3.6. Possible Mechanisms of Mitigation of IMI Toxic Effects by Probiotic

The effects of *Lactobacillus brevis* 47f on the histopathological index and morphometric measurements were clearly observed through PCA. The use of the probiotic resulted in a reduction of the toxic effects of IMI on the examined organs. It is suggested that the toxicity of insecticides, including IMI, is primarily due to the excessive production of reactive oxygen/nitrogen species during the biotransformation of the xenobiotic [101], which can damage the liver and other organs [102]. IMI also disrupts the animal endocrine system by interacting with receptors and mimicking or suppressing hormone actions [103]. The endocrine-disrupting effects of IMI have been demonstrated on the gonads of *Danio rerio* [104].

According to current understanding, the main mechanisms by which probiotic strains detoxify xenobiotics are as follows: (i) binding or adhesion of the xenobiotic to bacterial cells, (ii) direct chemical or physical biotransformation of the toxicant by the probiotic strain or other members of the microbiome, (iii) formation of stable bonds with metabolites of the bacterial community, (iv) modulation of the host’s ability to metabolize xenobiotics through the reduction of oxidative stress and stimulation of a nonspecific immune response, and (v) improvement of gut barrier function and peristalsis, which helps reduce toxin absorption [15]. In the case of IMI exposure, the probiotic could participate in the following stages of xenobiotic metabolism: during gastrointestinal entry and before absorption, and after metabolism in the liver, during which the chemical compounds are excreted through the kidneys/gills or with bile into the intestine [105]. 

The probiotic strain probably participated in the binding/metabolization of IMI and its metabolites, thereby reducing their entry into the excretory organs. In addition, the stabilization of the intestinal microbial community by the probiotic could contribute not only to the reduction of toxic effects but also to the enhancement of intestinal tissue regeneration (restoration of barrier function, modulation of the inflammatory response [5]) through the production of biologically active substances (endotoxins, short-chain fatty acids, secondary bile acids, liposaccharides) [96,106]. The inflammatory response observed in IMI-exposed groups can also be induced by endotoxins produced by the commensal microbiome [107]. Some gut histological changes observed in this study, such as increased numbers of goblet cells and intraepithelial leukocytes, may also be associated with shifts in microbiota composition, which is an essential component for proper immune system function [95,108,109]. 

In some studies, LAB have demonstrated high efficacy in the detoxification of various pollutants. This is attributed to their ability to modulate pro/antimicrobial signaling pathways and mucosal epithelial tight junction proteins [110,111], synthesize bioactive compounds that can bind to xenobiotics and reduce their toxicity [112], and enhance mucosal adhesion through competitive integration with mucin [59,113]. However, there is currently insufficient data on the metabolism of IMI in mammals and fish, which hinders clear conclusions about its detoxification mechanisms.

The study found that chronic exposure to low concentrations of imidacloprid leads to severe histological abnormalities in the intestines and kidneys, which can ultimately result in fish mortality. The introduction of *Lactobacillus brevis* 47f can alleviate some of the negative effects of IMI. To determine the mechanism of detoxification action of *Lactobacillus brevis* 47f, additional studies are needed, in which histological indicators of the intestines and kidneys can be used as target organs. This is partially supported by the results of the analysis of pro/anti-inflammatory gene expression, which showed a multidirectional pattern but indicated damage to intestinal cells and the possibility of their recovery.

## 4. Materials and Methods

### 4.1. Animal Collection and Maintenance

This study was conducted in accordance with the guidelines of the Local Ethics Commission of the Scientific and Technical Council of the Moscow State University of Technology and Management (approval number 8, 21 June 2022).

Four-month-old wild-type *Danio rerios* with an average size of 1.93 ± 0.11 cm and a weight of 0.21 ± 0.03 g were used for the experiment. They were kept in 300-L aquariums with systems of mechanical and biological filtration, with 10% water replacement per day. Prior to the experiment, the fish were fed with Tetra Min Flakes XL (Melle, Germany).

Healthy individuals without visible injuries were selected for the study. The experiment was conducted using 50-L tanks with constant aeration, housing 25 individuals of both sexes. The temperature (24 °C), light regime (12:12 h), and hydrochemical parameters (pH 7.2 ± 0.2; O_2_ 7.8 ± 0.3; NH_4_ < 0.05; NO_2_ 0.2 ± 0.01; NO_3_ 5.3 ± 1.1) were maintained according to the conditions in the housing aquariums. The experimental fish were fed ad libitum. 

### 4.2. Bacterial Strain

The *Lactobacillus brevis* 47f (BioSample ID: SAMN03470252) used in the study was first obtained from the feces of a healthy female living in the Central European part of the Russian Federation. It belongs to the collection of the Laboratory of Genetics of Microorganisms at Vavilov Institute of General Genetics, Russian Academy of Sciences. The strain has been deposited in the All-Russian Collection of Industrial Microorganisms (No. B-12237).

### 4.3. Culture Media, Growth Conditions and Lyophilization

The *Lactobacillus brevis* 47f were grown in liquid medium MRS (HiMedia, Mumbai, India), as well as on solid agar medium MRS (HiMedia, Mumbai, India) at 37 °C under oxygen-free conditions in a desiccator, oxygen in which was burned by the flame of a candle.

The MRS liquid medium contained 10.0 g/L proteose peptone, 10.0 g/L beef extract, 5.0 g/L yeast extract, 20.0 g/L dextrose, 1.0 g/L polysorbate 80, 2.0 g/L ammonium citrate, 5.0 g/L sodium acetate, 0.1 g/L magnesium sulfate, 0.05 g/L manganese sulfate, and 2.0 g/L dipotassium phosphate (pH 6.5 at 25 °C). The MRS solid agar medium contained 10.0 g/L proteose peptone, 10.0 g/L HM peptone B, 5.0 g/L yeast extract, 20.0 g/L dextrose (glucose), 1.0 g/L polysorbate 80 (Tween 80), 2.0 g/L ammonium citrate, 5.0 g/L sodium acetate, 0.1 g/L magnesium sulfate, 0.05 g/L manganese sulfate, 2.0 g/L dipotassium hydrogen phosphate, and 12 g/L agar (pH 6.5 at 25 °C).

For lyophilization, an 18-h strain culture (10^9^ Colony-forming unit/mL, CFU/mL) was centrifuged for 10 min at 7000× *g* at 4 °C, washed with PBS buffer (KH2PO4 1.7 mM, Na2HPO4 5.2 mM, NaCl 150 mM, pH = 7.4), resuspended in a lyophilization medium (10% sucrose, 1% gelatin), frozen at −20 °C during the day and dried for 48 h at –52 °C and 0.42 mBar on a Labconco 2.5 freeze dryer (Labconco, Kansas City, MO, USA). Vials were stored at +4 °C and the viability of the lyophilizates did not change over the course of one year. The viability and titer of lyophilisates were checked before using them in the experiment.

### 4.4. Experimental Design

Imidacloprid (*N*-{1-[(6-Chloro-3-pyridyl)methyl]-4,5-dihydroimidazol-2-yl}nitramidel (CAS no. 138261-41-3, 3Way Pharm Inc., Shanghai, China)) was used as a model toxicant. The experimental concentration of imidacloprid was chosen as 2500 µg/L in accordance with the established for danio LC50 [114]. This concentration could potentially be detected in the aquatic environment during accidental spills [50,115]. The Imidacloprid solution for the experiment was prepared immediately before putting it into the tanks, due to the rapid degradation of imidacloprid in the aquatic environment [115]. The water in the experimental groups was replaced every two days to ensure a stable concentration of the toxicant.

Four groups (in three replicates) were involved in the experiment: a control group (CTR) that received the base feed without any supplements, the imidacloprid group (CIM) that received the base feed while being exposed to imidacloprid solution, the *Lactobacillus brevis* 47f (LAC) group that received probiotic bacteria in the feed, and the imidacloprid + *Lactobacillus brevis* 47f (LIM) group that received the feed with probiotic while being exposed to imidacloprid solution. The duration of the chronic experiment was 60 days. During the experiment, daily survival was monitored, and weight parameters were measured at 0, 30, and 60 days of the experiment in 10 random individuals from each experimental group.

### 4.5. Feed Preparation

The commercial feed Coppens Scarlet 0.5–0.8 mm [protein 53%, fat 13%, fiber 0.7%, ash 8.8%] (Coppens, Helmond, The Netherlands) was used as the basic diet in the experiment. The probiotic preparation for the LAC and LIM groups was incorporated into the feed through encapsulation using sodium alginate (Ruskhim, Moscow, Russia). To perform this, alginate was dissolved in distilled water (0.5 g per 100 mL) [116], and then a freeze-dried culture of *Lactobacillus brevis* 47f at a concentration of 1 × 10^8^ CFU/g was added to the solution and thoroughly mixed. Subsequently, the prepared solution was evenly sprayed onto the feed using a sprayer with a high-pressure pump (Santrade, Markham, ON, Canada). The feed was then air-dried at 4 °C for 15 h and stored at 4 °C. The quality of the feed with the probiotic was monitored by sowing on a solid agar medium MRS (HiMedia, India). The minimum criterion for bacterial survival was 90% for two weeks. The same amount of sodium alginate was added to the control feed and the positive control feed. To preserve the probiotic properties and quality of the feeds, they were prepared anew for all groups every two weeks.

### 4.6. Histological Preparations

After the end of the experiment (on the 60th day), four fish without visible lesions were selected from each experimental group to make histological sections. Three sections were made from each selected fish (n = 4 × 3). The fish were anesthetized in an MS-222 solution (10 mg/L) and then fixed in Davidson’s solution for 24 h. Tissue samples were subsequently dehydrated in a series of graded alcohols and embedded in paraffin. Next, total serial sections (4 μm) were prepared in the frontal plane and stained with hematoxylin and eosin (H&E) and periodic acid-Schiff (PAS). The histological sections were prepared and stained following the method described by Suvarna et al. [117].

The histological preparations were examined using an Olympus BX53 light microscope (Olympus Corporation, Tokyo, Japan) with Carl Zeiss ERc 5s (Zeiss, Oberkochen, Germany) and ToupCam 16.0 MP (ToupTek Photonics, Hangzhou, China) ocular attachments. The ZEN lite software v3.6 (Zeiss, Oberkochen, Germany) and ToupCam view 16.0 (ToupTek Photonics, Hangzhou, China) were used for image acquisition.

### 4.7. Histomorphometric Analysis

Morphometric parameters of the intestine, liver, and kidney were measured using the ImageJ v1.53t software (Wayne Rasband, NIH, [118], Kensington, MD, USA). At least 15 measurements were taken for each preparation, depending on the specific morphometric parameter (n = ~15 × 4).

The following measurements were taken at random sections of the slides: the height of the epithelium (IEH), the width of the intestinal muscularis (MW), and thickness of distal and proximal tubules (WTD, WTP). A perpendicular line was drawn to the plane of the tissue base (serous membrane, basal membrane) for these measurements [119,120,121]. The width of the lamina propria (LPW) and liver sinusoid capillaries (SW) were measured by drawing two perpendicular lines in the direction plane of the histological structure being measured. The number of goblet cells (GCC) and intraepithelial leukocytes (IELC) were estimated per 100 μm of intestinal epithelium [39,121]. Goblet cells with distinct mucin secretion located in the apical part of the epithelial layer were counted (GCS). When counting intraepithelial leukocytes, cells near the base of the villi were not included in the count [119,122]. For liver morphometric indices (hepatocyte nuclei area [HNA]/perimeter [HNP]/diameter [HND], hepatocyte cell area [HA], hepatocyte cytoplasm area [HCA]), cells with a well-defined nucleus were recorded. The following parameters were measured in the kidney: glomerulus area (GA), corpuscle area (CA), Bowmans spaces area (BSA). The measured histomorphometric parameters and abbreviations used are shown in Figure S1.

### 4.8. Semi-Quantitative Assessment of Histological Lesions

Histopathological index (HI) was calculated according to Bernet et al. [123]. Briefly, the importance factor (w), which reflects the effects of histopathological changes in organ function and the ability of fish to survive each type of histopathological abnormality, varies from one to three. The investigated histological abnormalities are divided into four groups (reaction pattern): circulatory changes, regressive and progressive changes, and inflammatory response (Table S3). The value of each histopathological lesion (a) was assigned a range of zero to five, where zero represents the absence of changes on all examined slices and five represents the presence of changes on 80–100% of slices [27].

The index of each reaction pattern for the intestine, liver, and kidney of *Danio rerio* was calculated according to the Formula (1):

(1) $${HI}_{rp\;org}=\sum_{alt}\left(a*w\right)$$

where HIrp org is the reaction pattern index; a is the score value and w is the importance factor for each histopathological abnormality (Table S1). The factors importance for histopathological abnormalities were selected based on the works of other authors [27,35,36,39,123,124,125,126,127].

Next, the total response index for each organ (HI_ioI_, HI_ioL_, HI_ioK_) was calculated by adding the index values for each response pattern (HI_pcor_, HI_cdor_, HI_cdor_, HI_ioor_) (abbreviations presented in Table S4). The total histopathological index reflecting the total effect of the studied toxicant on vital functions of *Danio rerio*, based on histological disturbances, was calculated by adding the three indices of the total reaction of each organ.

### 4.9. RNA Extraction

After the end of the experiment (on the 60th day), intestinal tissues were sampled from three *Danio rerio* individuals from each experimental group. After centrifugation for 1 min at 12,000× *g*, the pre-cooled cells were treated by ExtractRNA (Eurogene, Moscow, Russia). Chloroform/isoamyl alcohol (24:1, *v*/*v*) was added to the selected supernatant, and then it was vortexed. After centrifugation for 15 min at 12,000× *g*, the upper phase was treated with water-saturated phenol (pH = 4.5–5) and was vortexed. The mixture was centrifuged for 15 min at 12,000× *g* until the interphase disappeared, the upper phase was selected, and chloroform was added. The mixture of isopropanol/High Salt Solution (0.8 M sodium citrate; 1.2 M NaCl) (9:1, *v*/*v*) was added to the aqueous phase (which previously was removed to a new Eppendorf). Then the mixture was incubated on ice and centrifugation for 30 min at 12,000× *g* was performed. After that, the sediment was washed with 75% ethanol and dissolved in water. The quantitative analysis of the isolated RNA was carried out using a Qubit fluorimeter (Invitrogen, Carlsbad, CA, USA).

### 4.10. Purification of RNA and Reverse Transcription Reaction

To remove the remaining genomic DNA, DNase I (TURBO DNA-free Kit, Ambion, Invitrogen, Carlsbad, CA, USA) was used according to the manufacturer’s recommendations. The cDNA synthesis reaction, using the isolated RNA matrix, was carried out using a commercial set “iScript Select cDNA Synthesis kit” (Bio-Rad, Hercules, CA, USA) according to the manufacturer’s instructions.

### 4.11. Real-Time DNA Amplification

A total of 1 ng of cDNA was used for real-time qPCR with the qPCRmix-HS SYBR kit (Evrogen, Moscow, Russia) on a CFX96 Touch machine (Bio-Rad, USA). Amplification reaction program: 95 °C–5 min, 95 °C–20 s, 56 °C–30 s, 72 °C–1 min, the last three stages–50 cycles, 72 °C–5 s. CFX Manager V 3.1 (Bio-Rad, USA) was used to analyze the qPCR results: relative normalized expression of three biological replicates was calculated as ΔΔCq and genes actb1 was used as reference [128]. The primers were picked by primer-BLAST for qPCR [129] (Table S5).

### 4.12. Statistical Analysis

Comparison data of the analyzed variables are presented as mean ± SD. Statistical significance was determined using nonparametric tests (Kruskal–Wallis test, Mann-Whitney U-test), according to the distribution of the data and the homogeneity of variances (assessed with the Shapiro-Wilk and Levene tests). *p*-value < 0.05 was considered statistically significant. Statistical analyses were conducted using GraphPad Prism version 9.0 software (GraphPad, San Diego, CA, USA) and R software (v3.5.2)/RStudio [130,131]. Principal component analysis (PCA) was performed to assess the relationship between the toxicant/probiotic treatment and the analyzed histological parameters.

## 5. Conclusions

Chronic exposure of *Danio rerio* to IMI solutions (2500 µg/L) for 60 days resulted in the development of several histopathological disorders in the kidneys and intestines, potentially leading to fish mortality. The kidneys showed signs of granulomatous inflammation upon exposure to the toxicant. In this study, *Lactobacillus brevis* 47f was demonstrated to mitigate the toxic effects of sublethal concentrations of imidacloprid on *Danio rerio*. It led to a decrease in the number of histological lesions in the intestines and kidneys, as well as exerted multidirectional effects on various pro/anti-inflammatory cytokines. These findings lay the groundwork for further investigation of *Lactobacillus brevis* 47f as a probiotic treatment that can alleviate the toxic effects of xenobiotics. The histological disturbances observed in this study also highlight the potential use of imidacloprid as a model toxicant at low (sublethal) concentrations, representing the neonicotinoid class of insecticides.

## Data Availability

Not applicable.

## Supplementary Materials

The following supporting information can be downloaded at: https://www.mdpi.com/article/10.3390/ijms241512290/s1.
