# Supplementary material for: Ability of Lactobacillus brevis 47f to Alleviate the Toxic Effects of Imidacloprid Low Concentration on the Histological Parameters and Cytokine Profile of Zebrafish (Danio rerio)
Source: Int J Mol Sci. 2023 Jul 31;24(15):12290. doi: 10.3390/ijms241512290 (PMC10418720; doi:10.3390/ijms241512290)
Supplement: Supplementary file 1 [file ijms-24-12290-s001.zip › ijms-2525717-supplementary.docx]

| 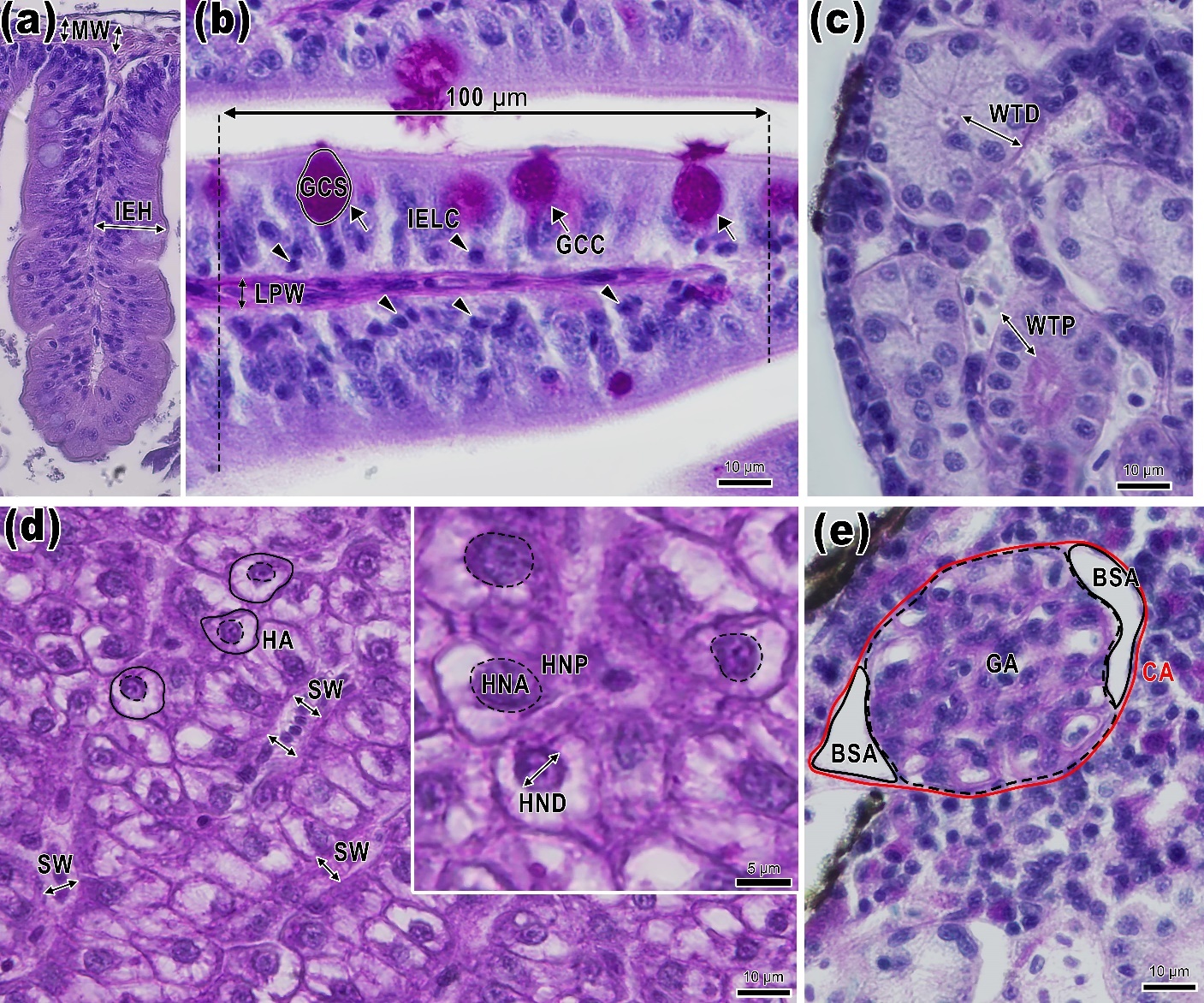 |
| --- |
| **Figure S1.** Histomorphometric parameters of *Danio rerio* measured in the study were as follows: (**a, b**) Intestine: Height of intestinal epithelium (IEH), Area of goblet cells (GCS), Thickness of the muscle layer (MW), Width of lamina propria (LPW), Number of goblet cells per 100 μm (two-way arrow) of epithelium (GCC, black arrows), Number of intraepithelial leukocytes per 100 μm of epithelium (IELC, black arrowheads); (**d**) Liver: Hepatocyte nuclei area (HN, dotted line), Hepatocyte nucleus perimeter (HNP), Hepatocyte nucleus diameter (HND), Hepatocyte area (HA, black line), Hepatocyte cytoplasm area (HCA), Nucleus/cytoplasm area ratio (NCR), Sinusoid capillary width (SW); (**c, e**) Kidneys: Glomerulus area (GA, dotted line ), Corpuscle area (CA, red line), Bowman space area (BSA, black line), Proximal tubule wall thickness (WTP), Distal tubule wall thickness (WTD). The scale bar represents 10 μm |

**Table S1**. Histopathological indices of intestine, liver, and kidney of experimental *Danio rerio*.

| **Alteration** | **CTR** | | **CIM** | **LAC** | | **LIM** |
| --- | --- | --- | --- | --- | --- | --- |
|  | Intestince | | | | | |
| Inflammatory responses | 2.5±1.65 | 7.5±0.86 | | 1±1 | 4.5±0.86 | |
| Progressive changes | 1±1 | 0.75±0.43 | | 0.75±0.82 | 1±0.7 | |
| Circulatory disorders | 0.25±0.43 | 1.5±0.5 | | 1±0.7 | 1±0 | |
| Regressive changes | 2±1.22 | 3.75±1.78 | | 2±1.22 | 1.25±1.08 | |
| Organ index | 5.75±2.38 | 13.5±2.29 | | 4.75±2.58 | 7.75±1.92 | |
|  | Liver | | | | | |
| Inflammatory responses | 0.75±0.82 | 1.25±0.82 | | 1.75±1.08 | 1±1 | |
| Progressive changes | 0.5±0.86 | 2.5±0.5 | | 0.5±0.5 | 1.5±0.5 | |
| Circulatory disorders | 0.25±0.43 | 0.5±0.5 | | 0.25±0.43 | 1.25±0.43 | |
| Regressive changes | 1±1 | 5.75±1.29 | | 1±1 | 3±1.22 | |
| Organ index | 2.5±0.5 | 10±2.44 | | 3.5±0.5 | 6.75±1.78 | |
|  | Kidney | | | | | |
| Inflammatory responses | 0.75±0.43 | 0.5±0.5 | | 0.5±0.86 | 0.25±0.43 | |
| Progressive changes | 1±0 | 1.25±0.43 | | 1±1 | 1±1 | |
| Circulatory disorders | 0±0 | 0.5±0.5 | | 0±0 | 0.5±0.5 | |
| Regressive changes | 3.5±1.5 | 12.25±1.08 | | 3.25±1.78 | 9±1.22 | |
| Organ index | 5.25±1.08 | 14.5±1.65 | | 4.75±2.48 | 10.75±1.63 | |
|  | | | | | | |
| Total index | 13.5±3.04 | 36.75±5.67 | | 13±4.52 | 25.75±4.54 | |

**Table S2**. Morphometric parameters of intestine, liver, and kidney of experimental *Danio rerio.*

| **Morhometric parametr** | **Experemental group** | | | |
| --- | --- | --- | --- | --- |
|  | **CTR** | **CIM** | **LAC** | **LIM** |
| Intestine | | | | |
| Intestine epithelium high | 30.71±1.29 | 25.79±0.9 | 28.56±2.86 | 32.56±2.96 |
| Goblet cell square | 81.18±3.42 | 78.83±13.77 | 80.22±8.88 | 84.96±7.07 |
| Lamina propria wide | 6.32±0.96 | 9.93±1.58 | 10.22±0.91 | 8.63±0.61 |
| Muscularis wide | 11.71±0.78 | 12.59±3.13 | 14.6±2.73 | 11.57±1.01 |
| Goblet cell count per 100 μm | 2.69±0.16 | 5.03±0.87 | 8.33±1.4 | 2.85±0.74 |
| Intraepithelial leucocyte count per 100 μm | 8.93±0.16 | 10.76±0.63 | 8.38±0.77 | 10.55±0.56 |
| Liver | | | | |
| Hepatocyte nuclei area | 17.18±1.23 | 23.07±2.12 | 18.82±1.58 | 19.06±0.89 |
| Hepatocyte nuclei perimeter | 15.78±1.01 | 17.09±1.22 | 16.53±1.17 | 16.08±0.4 |
| Hepatocyte nuclei dimeter | 9.34±0.35 | 10.03±0.72 | 10.85±1.25 | 9.34±0.19 |
| Hepatocyte cytoplasm area | 68.07±5.31 | 62.54±3.71 | 59.29±6.54 | 67.4±5.3 |
| Nuclei/cytoplasm ratio | 0.26±0 | 0.38±0.05 | 0.34±0.03 | 0.3±0.02 |
| Sinusoid wide | 5.54±0.86 | 6.41±0.5 | 5.91±0.28 | 6.47±0.22 |
| Kidney | | | | |
| Area glomerulus | 974.98±74.21 | 1037.8±45.44 | 974.34±98.54 | 962.18±38.1 |
| Area corpuscle | 1320.21±79.72 | 1701.6±105.3 | 1361.5±101.6 | 1489.5±72.9 |
| Area Bowmans space | 345.22±30.68 | 663.77±62.87 | 387.24±11.97 | 527.33±70.2 |
| Thickness wall proximal tubulesc | 9.04±0.69 | 10±0.47 | 8.5±0.61 | 8.94±0.44 |
| Thickness wall distal tubules | 11.75±0.85 | 10.44±0.78 | 9.14±0.43 | 9.96±1.14 |

**Table S3.** Histopathological lesions in the intestines, liver, and kidneys of *Danio rerio* and their importance factor (w).

| **Reaction pattern** | **Histopathological lesion** | **w** |
| --- | --- | --- |
|  | Liver | |
| Circulatory disorders | Vasodilation | 1 |
|  | Sinusoidal congestion | 1 |
| Regressive changes | Necrosis | 3 |
|  | Vacuolization (Steatosis) | 1 |
|  | Pyknotic nuclei | 2 |
| Progressive changes | Hypertrophy of hepatocyte nucleus | 1 |
|  | Hypertrophy of hepatocyte cytoplasm | 1 |
| Inflammatory responses | Mononuclear cell infiltrates | 2 |
|  | Aggregates of macrophages | 2 |
|  | Melanomacrophages | 1 |
|  | Intestince | |
| Circulatory disorders | Haemorrhage/hyperaemia/aneurysm | 1 |
|  | Intercellular oedema | 1 |
| Regressive changes | Necrosis | 3 |
|  | Irregular cell appearance | 1 |
|  | Disrupted myofibril architecture/structure of smooth muscle fibers | 1 |
| Progressive changes | Supranuclear vacuoles | 1 |
|  | Hypertrophy of mucous cell | 1 |
|  | Hypertrophy of epithelial cells | 1 |
| Inflammatory responses | Presence of EGC | 2 |
|  | Infiltration of mononuclear leucocyte | 2 |
|  | Kidney | |
| Circulatory disorders | Hemorrhage | 1 |
| Regressive changes | Necrosis of visceral (podocytes) layer | 3 |
|  | Necrosis of parietal layer | 3 |
|  | Melanomacrophages center necrosis | 2 |
|  | Vacuolation of pseudostratified mucus epithelium | 1 |
|  | Increase in Bowman’s space | 1 |
|  | Necrosis of tubule | 3 |
|  | Vacuolation of glomerulus | 1 |
|  | Pyknotic nucleus of hematopoietic tissue | 2 |
| Progressive changes | Hypertrophy | 1 |
|  | Hyperplasia of hematopoietic tissue | 1 |
|  | Hypertrophied cells and narrowing of the tubular lumen | 1 |
|  | Hypertrophy of epithelial cell | 1 |
| Inflammatory responses | Edema | 1 |

**Table S4**. Abbreviation of histopathological indexes and morphometric parameters used in the work.

| **Abbreviation** | **Deciphering** |
| --- | --- |
| HI_irI_ | Histopathological index inflammation response intestine |
| HI_pcI_ | progressive changes intestine |
| HI_cdI_ | circulatory disorders intestine |
| HI_rcI_ | regressive changes intestine |
| HI_ioI_ | organ index Interstice |
| IEH | intestine epithelium high |
| GCS | goblet cell area |
| MW | muscularis wide |
| LPW | lamina propria wide |
| GCC | goblet cell count |
| IELC | intraepithelial leucocyte count |
| HI_irL_ | Histopathological index inflammation response liver |
| HI_pcL_ | progressive changes liver |
| HI_cdL_ | circulatory disorders liver |
| HI_rcL_ | regressive changes liver |
| HI_ioL_ | organ index liver |
| HNA | hepatocyte nuclei area |
| HNP | hepatocyte nuclei perimeter |
| HND | hepatocyte nuclei dimeter |
| HA | hepatocyte cell area |
| HCA | hepatocyte cytoplasm area |
| NCR | nuclei/cytoplasm ratio |
| SW | sinusoid wide |
| HI_pcK_ | Histopathological index inflammation response kidney |
| HI_cdK_ | progressive changes kidney |
| HI_rcK_ | circulatory disorders kidney |
| HI_ioK_ | regressive changes kidney |
| HI_ioK_ | organ index kidney |
| GA | glomerulus area |
| CA | corpuscle area |
| BSA | Bowmans spaces area |
| WTP | wall thickness proxisomal tube |
| WTD | wall thickness distal tube |
| HITO | Total histopathological index |
| SR | survival rate 60 days |

**Table S5.** Pro/anti-inflammatory *Danio rerio* primer sequence used for qPCR.

| **Target** | **R/F** | **Sequences (5-3)** | **NCBI access number** |
| --- | --- | --- | --- |
| Interleukin 1 beta (IL-1B) | F | CATTTGCAGGCCGTCAC | NM_212844.2 |
|  | R | GGACATGCTGAAGCGCACT |  |
| Tumour necrosis factor α (TNF-α) | F | GCTGGATCTTCAAAGTCGGGTGT | NM_212859.2 |
|  | R | TGTGAGTCTCAGCACACTTCCAT |  |
| Interferon phi 1 (ifnphi1) | F | GAATGGCTTGGCCGATACAGGAT | NM_207640.1 |
|  | R | TCCTCCACCTTTGACTTGTCCAT |  |
| Interleukin 6  (IL-6) | F | TCAACTTCTCCAGCGTGAT | NM_001261449.1 |
|  | R | TCTTTCCCTCTTTTCCTCCT |  |
| Interleukin 8  (IL-8) | F | GTCGCTGCATTGAAACAGA | XM_001342570.7 |
|  | R | CTTAACCCATGGAGCAGAG |  |
| Interleukin 10  (IL-10) | F | CCCTATGGATGTCACGTCAT | NM_001020785.2 |
|  | R | CATATCCCGCTTGAGTTCCT |  |
| βactin (actb1) | F | ATGGATGAGGAAATCGCTGCC | NM_131031.2 |
|  | R | CTCCCTGATGTCTGGGTCGT |  |
